# Oral Anti-Tumour Necrosis Factor Domain Antibody V565 Provides High Intestinal Concentrations, and Reduces Markers of Inflammation in Ulcerative Colitis Patients

**DOI:** 10.1038/s41598-019-50545-x

**Published:** 2019-10-01

**Authors:** Suhail Nurbhai, Kevin J. Roberts, Timothy M. Carlton, Luana Maggiore, Marion F. Cubitt, Keith P. Ray, Jill Reckless, Hafeez Mohammed, Peter Irving, Thomas T. MacDonald, Anna Vossenkämper, Michael R. West, Gareth C. Parkes, J. Scott Crowe

**Affiliations:** 1 VHsquared Ltd., 1 Lower Court, Copley Hill, Cambridge Road, Babraham, Cambridge CB22 3GN UK; 2 VHsquared Ltd., Wellcome Sanger Institute, Wellcome Genome Campus, Hinxton, CB10 1SA UK; 3 RxCelerate Ltd. Babraham Research Campus, Cambridge, CB22 3AT UK; 4 Guy’s and St Thomas’ Hospital, London, UK; 5 Blizard Institute, Barts and the London School of Medicine and Dentistry, Queen Mary University of London, London, UK; 6 Royal London Hospital, London, UK

**Keywords:** Crohn's disease, Crohn's disease

## Abstract

V565 is an engineered TNFα-neutralising single domain antibody formulated into enteric coated mini-tablets to enable release in the intestine after oral administration as a possible oral treatment for inflammatory bowel disease (IBD). Following oral administration, ileal recovery of V565 was investigated in four patients with terminal ileostomy. Intestinal and systemic pharmacokinetics were measured in six patients with Crohn’s disease and evidence of target engagement assessed in five patients with ulcerative colitis. Following oral administration, V565 was detected at micromolar concentrations in ileal fluid from the ileostomy patients and in stools of the Crohn’s patients. In four of the five ulcerative colitis patients, biopsies taken after 7d dosing demonstrated V565 in the lamina propria with co-immunostaining on CD3^+^ T-lymphocytes and CD14^+^ macrophages. Phosphorylation of signalling proteins in biopsies taken after 7d oral dosing was decreased by approximately 50%. In conclusion, enteric coating of V565 mini-tablets provided protection in the stomach with gradual release in intestinal regions affected by IBD. Immunostaining revealed V565 tissue penetration and association with inflammatory cells, while decreased phosphoproteins after 7d oral dosing was consistent with V565-TNFα engagement and neutralising activity. Overall these results are encouraging for the clinical utility of V565 in the treatment of IBD.

## Introduction

Inflammatory bowel disease (IBD) is a chronic immuno-inflammatory condition of the GI tract comprising mainly Crohn’s disease (CD) and ulcerative colitis (UC). CD and UC each affect approximately 200 adults per 100,000 population and are major causes of morbidity and health care utilisation. While the exact causes of CD and UC are unknown, tumour necrosis factor alpha (TNFα) is a master pro-inflammatory cytokine in the pathogenesis^1^. An increased level of TNFα in the lamina propria of the intestinal tract mucosa may drive the initiation and perpetuation of the chronic inflammatory processes that damage intestinal epithelial cells, resulting in the loss of mucosal barrier integrity^2^ and contributing to the breakdown of intestinal immune homeostasis^3,4^.

Antibodies neutralising TNFα are highly effective for the treatment of IBD^5–8^ but currently these biological agents are administered parenterally and distributed systemically. This route of administration is inconvenient and can be uncomfortable, and there are also safety concerns associated with long term systemic suppression of the immune system, including risk of opportunistic infections and neoplasia, particularly in younger patients^9–11^. Oral administration, delivering anti-TNFα activity directly to the site of TNFα overproduction in the intestinal mucosa of IBD patients, could provide significant advantages to currently available therapeutic options. A key determinant of efficacy for orally delivered anti-TNFα antibodies is the ability to deliver adequate concentrations of active agent to the intestinal mucosa^12–14^. Recent *in vitro* studies suggest that proteases present in IBD colonic mucosal tissue contribute to a loss of the integrity and TNFα-neutralising activity of conventional antibodies including infliximab and adalimumab^15^. A recent study testing the bovine colostrum polyclonal anti-TNFα product AVX-470 in patients with UC delivered TNFα binding activity in the intestinal lumen after oral dosing and resulted in some positive trends in the assessment of clinical, endoscopic and biomarker endpoints suggesting that topical exposure to anti-TNFα might be therapeutically effective^16,17^. An oral domain antibody optimised for resistance to intestinal and inflammatory proteases and thereby able to deliver high concentrations of active neutralising antibody to the site of inflammation would have increased potential for neutralisation of TNFα in the mucosa.

V565 is a 12.6 kDa anti-TNFα heavy chain variable domain antibody isolated from a phage library produced from lymphocytes of a human TNFα hyperimmunised llama and engineered to be resistant to intestinal and inflammatory proteases. V565 has been demonstrated to have excellent survival in the intestinal tract of animals and humans^18,19^. V565 has similar potency to adalimumab in neutralising both membrane and soluble TNFα, and inhibits the production of cytokines from human UC biopsies to a similar degree and with a similar pattern to infliximab^18^. The present series of experiments was designed to demonstrate that after oral dosing to human subjects including patients with CD and UC, V565 would be present in high concentrations in the intestinal lumen, enter the site of inflammation in the intestinal mucosa, bind to and neutralise TNFα, and reduce inflammatory processes.

## Materials and Methods

### Study populations, ethical approvals and informed consent

Three sets of human volunteers were recruited for study. All aspects of the protocols were reviewed and approved by relevant ethics committees. All subjects provided written informed consent. Where dose escalation was included as part of the study plan a safety and dose escalation committee reviewed all data at least 24 hours prior to next dosing, and issued explicit approval for a higher dose to be used. Subjects were not permitted to enter if they had any contra-indications to the use of an anti-TNFα antibody or if, in the opinion of the investigator, they had any other medical condition which would hinder their ability to comply with study procedures or the interpretation of results. Research ethics committee approval (reference 10/H0704/73) for studies using human tissue was obtained from the NRES Committee London – City & East. The study was also approved by the local Barts and The London School of Medicine and Dentistry QMUL Joint R&D office. For ileostomy patients and CD patients, the study protocol, written study subject information, informed consent form (ICF), and any other appropriate study-related information were reviewed and approved by the Office for Research Ethics Committees Northern Ireland (ORECNI), Lisburn, Co. Antrim BT28 1TW. For UC patients, the protocol was reviewed and approved by the East of England - Cambridgeshire and Hertfordshire Research Ethics Committee, Nottingham NG1 6FS. All aspects of the work described have been conducted following Good Clinical Practice and Good Clinical Laboratory Practice guidelines.

To evaluate ileal V565 concentrations after oral dosing, four patients with a terminal ileostomy but no history of CD were selected for study. The ileostomy must have been for non-malignant disease and had to have been present for at least 18 months. Each subject took a single oral dose of 1665 mg V565 following which ileostomy bags were collected hourly for the first 12 hours, and then at 16 hours, 20 hours and 24 hours after dosing. The contents of bags were analysed for concentration of V565.

To evaluate faecal V565 concentrations after oral dosing, six patients with Crohn’s disease for a minimum of six months were selected for study. Prior to dosing, the diagnosis of CD was confirmed by a gastroenterologist and a clinical assessment of severity was carried out. As this was primarily a pharmacokinetic study, patients were excluded if they required surgery, had a current abscess, a non-inflammatory stricture, or a history of obstruction. The first two subjects took a single oral dose of 555 mg V565. Following review by the safety/dose escalation committee four further subjects took a single oral dose of 1665 mg V565. For both the determination of ileal fluid concentrations and faecal concentrations, samples were immediately frozen at −80 °C to prevent degradation of V565 prior to analysis. Upon thawing for analysis, protease inhibitors were added to prevent further degradation in the sample. Serial blood samples were taken to assess systemic V565 exposure, and all stool samples were collected for up to three days after dosing for detection of faecal V565. In addition, urine was collected from all patients over periods 0–4 h, 4–8 h, 8–12 h and 12–24 h post dose.

To demonstrate passage of V565 into inflamed tissue and reduction of inflammation after oral dosing, five patients with an established diagnosis of UC present for at least 12 months were recruited. They needed to have mild to moderate disease defined as a Mayo score of between 3 and 10 including an endoscopic sub-score of at least 1. Patients with isolated proctitis were excluded from this study. After initial flexible sigmoidoscopy with biopsy all subjects were provided with oral V565 to be taken at a dose of 555 mg tid as an out-patient for seven days. At the end of the dosing period all patients returned for repeat sigmoidoscopy and biopsies. Biopsies were taken at the same distance from the anal verge as pre-dose biopsies. Biopsies were analysed for phosphoprotein levels as described by Vossenkämper *et al*.^20^ and examined for V565 presence using immunohistochemistry. Serum, urine and faecal samples were taken from all subjects both pre- and post-dosing for assessment of V565 pharmacokinetics. Blood samples were also taken for anti-drug antibody (ADA) assessment.

### Reagents and antibodies

V565 is an engineered 115 amino acid 12.6 kDa single variable domain antibody, which neutralises soluble and membrane bound human TNFα with potency similar to adalimumab and infliximab^18^. ID2A is a control variable domain antibody that was raised against a non-immune target. Both domain antibodies were isolated from hyper-immunised llama phage libraries, engineered for protease stability, produced from *Saccharomyces cerevisiae* and purified using CaptoS ion-exchange chromatography. For the phase I ileostomy and Crohn’s volunteers the drug substance was produced at Lonza, Kourim, Czech Republic. For the UC study drug substance was produced at Fujifilm, Billingham, UK. All drug product, mini-tablet and capsule production was undertaken by Quay Pharma, Deeside, UK.

### Study drug formulation

The drug substance V565, is formulated into 3 mm round mini-tablets. The mini-tablets are coated with a polymer sub-coat and a Eudragit® enteric coat is applied to enable release in the required area of the intestinal tract. Once coated, the mini-tablets are encapsulated in hydroxypropyl methylcellulose (HPMC) capsules. The formulation is engineered to release the mini-tablets from the capsules in the stomach. The size of the mini-tablets enables them to escape through the pylorus intact and migrate into the intestinal tract. The mini-tablet coating releases active molecule at a pH designed to ensure delivery of high concentrations of V565 to the ileum and distal parts of the GI tract^19^.

### Study 1: Assessment of oro-ileal recovery of V565

One female and three male subjects were entered into the study. They were aged between 31 and 59 years old. Three of them had ulcerative colitis for between 11 and 40 years and the fourth had a prior intestinal stricture. Ileostomies had been in place for between 1 and 38 years. Each patient received 9 capsules, each containing 15 mini-tablets of V565, giving a total dose of 1665 mg.

The contents of the ileostomy bags at each time point were examined, and intact or partially dissolved mini-tablets were removed and dissolved completely in PBS (pH 7.3) containing 0.1% BSA. Ileal fluid samples were centrifuged and supernatants were removed. V565 concentrations in ileal fluid supernatants and in solutions from the mini-tablet dissolutions were measured using a biotinylated adamilumab competition ELISA, whereby biotinylated adalimumab-TNFα binding was prevented in a V565 concentration dependent manner^18^.

### Study 2: Assessment of V565 concentrations in faecal, urine and blood samples

Four female and two male subjects were entered into the study. They were aged between 26 and 65 years old and had been diagnosed with CD for between 1 and 15 years. As this was a pharmacokinetic study there was no formal assessment of disease severity. All patients were assessed by a Consultant Gastroenterologist as having mild CD at the time of study entry.

Faecal samples were stored at −80 °C until processing. Faeces were weighed, mixed 1:4 (w/v) with ice-cold assay buffer (0.1% BSA, 0.6 M NaCl, 0.05% Tween 20, 0.5% human AB serum and 2x working concentration of SigmaFast protease inhibitors in PBS), then homogenised and centrifuged after which the supernatants were removed and diluted for V565 analysis using the biotinylated adalimumab competition ELISA. V565 concentrations in the faeces were determined using the V565 faecal supernatant concentrations and the dilution factor used in the faecal slurry preparation. It was assumed that the specifc gravity of faeces is 1 and that V565 is freely diffusible throughout. V565 concentrations in serum and urine samples were measured using a similar, validated biotinylated adalimumab competition ELISA.

### Study 3: Assessment of V565 tissue localisation and activity

Seven patients were screened for the study. Two patients failed screening (history of melanoma; *Clostridium difficile* infection) and five male patients were entered into the study between June and October 2017. Dosed patients were aged between 27 and 44 years old and had been diagnosed with UC for between 1 and 23 years. All patients had mild to moderate disease: the Mayo score at the time of entry into study ranged between 3 and 7. Four of the five patients were on medication for UC (aminosalicylates, mercaptopurine, azathioprine) but there were no changes in any relevant medications during the course of the study. A summary of the patient characteristics is presented in Supplementary Table S1.

### Sample collection

Before the start of V565 dosing (Visit 2), 10 biopsies were taken from each subject from the area of the colon worst affected by disease. There was no standardisation of the distance from the anal verge for taking biopsies across all patients as it was considered more important to ensure biopsies were from inflamed tissue to facilitate the measurement of biological effect in each individual after dosing. Following dosing with V565 for 7d (Visit 3), six biopsies were taken from the same affected area from each subject and, to the extent possible, from the contralateral side of the colon (i.e. if the pre-dose biopsies were taken from the anterior wall the post-dosing biopsies were taken from the posterior wall at the same distance from the anal verge). Prior to dosing at Visit 2, and at Visit 3, samples of the colonic luminal contents (collected post enema via aspiration during sigmoidoscopy), serum and urine were collected for the measurement of V565 levels; serum samples were also taken for the assessment of systemic cytokine levels and C-Reactive Protein (CRP) levels.

### Assessment of V565 tissue localisation

For each subject at both Visits 2 and 3, four biopsies were embedded in Optimal cutting temperature compound (OCT) and frozen at −80 °C for immunohistological evaluation. Biopsy sections were fixed on ice for 10 min with 4% paraformaldehyde, then rinsed and air-dried. Sections were stored at −20 °C until analysed. A rabbit anti-V565 polyclonal antibody (SY7915, Eurogentec, custom preparation), followed by goat F(ab’)2 anti-rabbit, Alexa 594 (ThermoFisher scientific, Cat No. A11072) were used for immuno-localisation of V565 and were made up in PBS containing 0.6 M NaCl, 5% Milk (Marvel Premier Foods), 1% BSA and 0.05% Tween 20, and this solution with 5% goat serum and 5 µg/ml Hoechst 33342, respectively. In addition, anti-CD14 (FITC mouse anti human CD14, Biolegend 325603) and anti-CD3 (Alexa Fluor® 488 Mouse anti-human CD3, Biolegend 317310) were used for locating CD14 positive and CD3 positive cells respectively. The CD3 signal was enhanced by using goat anti-mouse Alexa Fluor® 488 (Abcam, ab150117). Slides were viewed using an Olympus AX70 microscope and images were captured sequentially for each flurophore (Alexa Fluor® 488, 594 and UV) using Image Pro-Plus (v7.0, Media Cybernetics). Exposure levels were set for each control section that had been treated with vehicle alone. At least three random fields of view were captured for each section for each biopsy. Images were coded such that subject number and pre- and post-V565 dosing were not identifiable, and scoring of V565 intensity was carried out on randomised images. A V565 fluorescence intensity score ranging from 1–5 was assigned to each field with 5 being the highest level of fluorescence.

### Processing and analysis of biopsies

#### Analysis of phosphoproteins

Following collection from each subject on Visit 2, two of the pre-dose biopsies were immediately snap-frozen and stored for subsequent analysis of phosphoprotein levels. Four biopsies were cultured (two biopsies per well) in 24-well plates (VWR International, Lutterworth, UK) in 300 μl serum-free HL-1 medium (Lonza) supplemented with glutamine, 100 U/ml penicillin, 100 μg/ml streptomycin, 50 µg/mL gentamicin at 37 °C, 5% CO_2_. Two biopsies were maintained in organ culture (OC) for 24 h with the addition of ID2A (negative control VHH) 150 nM and two with V565 150 nM. The tissue samples collected from each well at the end of the incubation period were snap-frozen and stored at −80 °C. On Visit 3 after 7d dosing with V565, two of the biopsies from each subject were immediately snap frozen and stored at −80 °C. For the analysis of phosphoprotein content, biopsies were thawed and lysed in RIPA Buffer (Sigma-Aldrich, St. Louis, MO) supplemented with phosphatase inhibitor cocktail 2 (Sigma-Aldrich) and protease inhibitor cocktail (Sigma-Aldrich), both at 1%. Protein concentrations of the lysates were determined by the Bio-Rad Bradford Protein assay (Bio-Rad Laboratories, Hemel Hempstead, UK) and samples diluted to 1.0 mg/ml in Array Diluent Buffer. The phosphorylation status of receptor tyrosine kinases (RTK) and signalling molecules was determined using PathScan RTK signalling arrays (Cell Signalling Technology, Danvers, MA), which detect 28 receptor tyrosine kinases and 11 important signalling nodes, when phosphorylated at tyrosine or other residues. A total of 150 μg protein of tissue lysate was probed onto an array, and the phosphoprotein levels were detected according to the manufacturer’s instructions. The chemiluminescent signals were detected on X-ray films, and the spot pixel intensities were measured using ImageJ software^20^.

### Additional exploratory biological measures

#### Analysis of serum samples

Serum samples for the measurement of cytokines and CRP concentrations were collected prior to dosing at Visit 2, and after the final dose at Visit 3. The concentrations of IL-6, IL-10 and TNFα were determined at the Immunoassay Biomarker Core Laboratory, University of Dundee, on a Simoa HD-1 analyser using the Quanterix^TM^ Simoa Human Cytokine 3-plex A according to the manufacturer’s instructions. Lower limits of quantification (LLOQ) for these cytokines were ≤0.05 pg/ml. CRP concentrations were determined by Safety Laboratory Services at the ACM Global Central Laboratory, York, UK.

#### Measurement of V565

Determination of V565 in human serum, urine and colonic lumen aspirate samples was performed at LGC Ltd, Fordham, UK. V565 concentrations were measured using a competition ELISA with biotinylated adalimumab on TNFα-coated plates LLOQ for this assay was 62.5 ng/ml for urine and serum, and 625 ng/ml for colonic lumen aspirate.

#### Determination of anti-V565 antibodies

Serum samples were collected on Visit 2 (pre-dose) and Visit 4 (14 days after the completion of dosing) for the assessment of anti-V565 antibodies (ADAs). Analysis of the samples was performed at LGC Ltd, using a semi-homogeneous immunogenicity assay. The positive control antibody used was an affinity purified rabbit polyclonal anti-V565, SY7915/6 from Eurogentec, Liège, Belgium. The sensitivity of this assay (LLOQ) for detection of a control anti V565 antibody was 52.5 ng/ml and drug tolerance at this level of ADA was ≤60 ng/mL V565. Drug tolerance at higher levels of ADA was ≤600 ng/mL V565.

Briefly, serum samples or controls were incubated with biotin-labelled V565 and ruthenium-labelled V565. The labelled V565 and ADAs (if present) formed a complex and upon addition to a streptavidin-coated MSD plate the complex bound to the streptavidin via the biotin labelled V565. After this incubation step a “read buffer” was added to the wells and the light emission measured using Sector Imager 6000 instrument. The intensity of the light signal emitted was proportional to the amount of anti-V565 antibody, or positive control antibody, in the sample.

### Assessment of safety

In all groups of patient volunteers a safety follow-up visit was performed 7–14 days after the last dose of study medication. This included evaluations of adverse events (AEs), clinical laboratory results (including ADAs), vital signs, physical examinations, ECGs and concomitant medications.

## Results

### Ileal fluid concentrations

**Figure 1:**
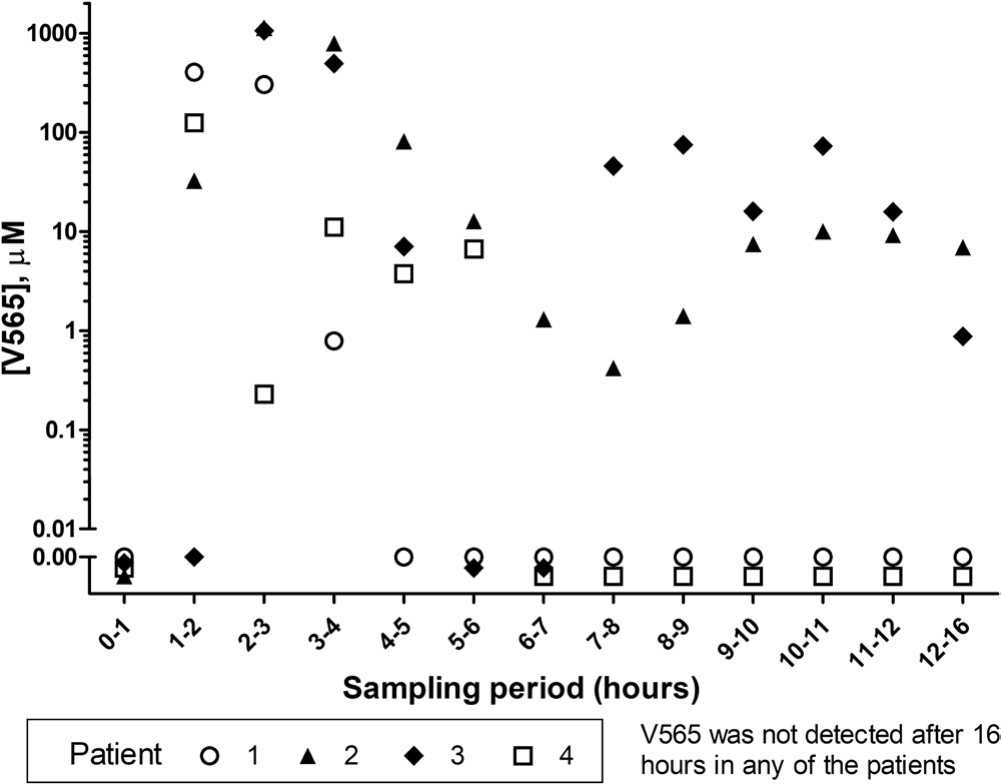
Analysis of V565 in ileal fluid samples taken after oral dosing of ileostomy patients. Ileal fluid samples were taken at specific time intervals from four ileostomy patients, given a single dose of 1665 mg V565; the V565 concentrations were measured by ELISA. The chart represents all observed data. The lower limit of quantification (LLOQ) was 49.6 nM. For reference, in a different study (Ungar *et al*.; 2016)^21^ the serum infliximab and adalimumab concentrations required for mucosal healing in Crohn’s patients were estimated to be in the range 40–80 nM when using a different assay.

Active, TNFα-neutralising V565 was found in the ileostomy bags of all four subjects, with particularly high concentrations in the 1–4 h samples and in two subjects V565 was detected 16 hours after dosing. The varied pattern of V565 detection in the ileal fluids was probably a reflection of the mini-tablet release pattern from the stomach. If release was synchronous, a V565 bolus would be detected in the ileal fluid but with no more being measured after the bolus passed. The V565 ileal fluid profile of patient 1 is consistent with this pattern. On the other hand, if mini-tablet release occurred in phases at different times, V565 might be found in ileal fluid over two periods, separated by a gap, as observed for patient 3. No V565 was found in any ileal fluid samples taken at greater than 16 h post V565 dosing (Fig. 1). In addition to the measured concentrations in ileal fluid, partially dissolved mini-tablets were recovered from all four subjects. In a post-hoc analysis of subjects 2–4 the quantity of V565 retained in these mini-tablets was assessed. The total recovery of V565 ranged between 66–82% of the administered dose.

### Pharmacokinetics in patients with Crohn’s disease

**Figure 2:**
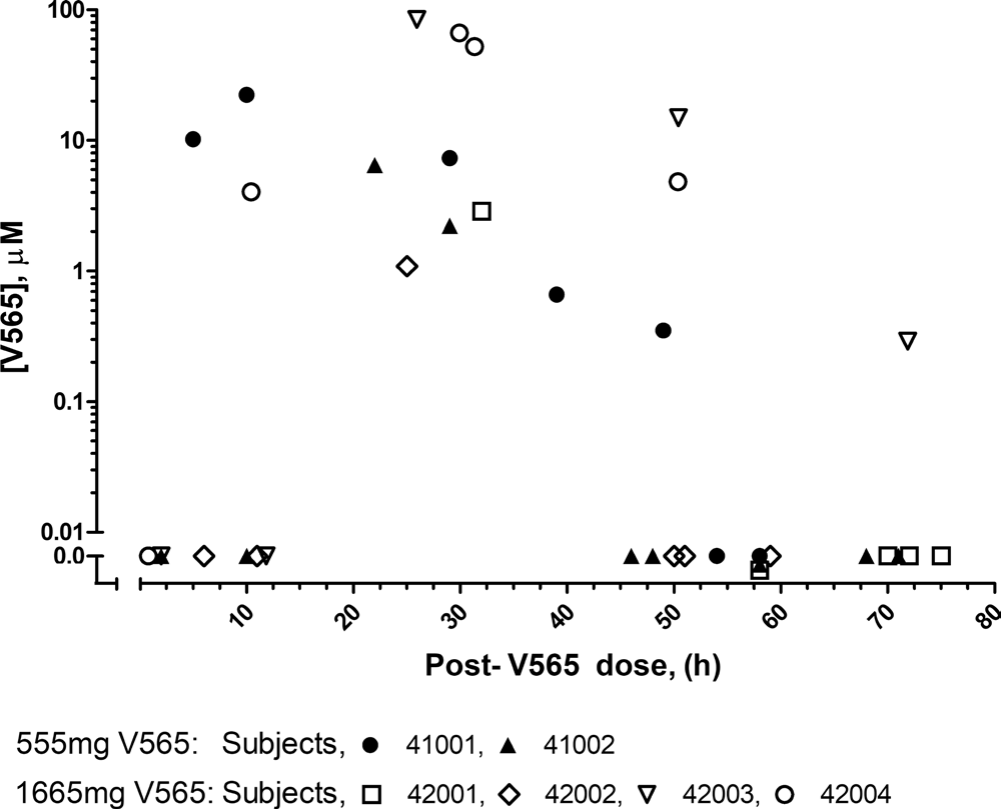
Crohn’s patients’ faecal V565 concentrations after single dose of either 555 mg or 1665 mg of V565. Six Crohn’s patients were given a single dose of V565. Faeces were collected up to 3 days after V565 administration from which faecal supernatants were prepared and analysed for active V565. V565 concentrations within the faeces were determined from the dilution factor used in preparing the faecal supernatants and the faecal supernatant V565 concentration, assuming that V565 was freely permeable throughout faeces and that faeces have a specific gravity of 1. For reference, in a different study (Ungar *et al*.; 2016)^21^ the serum infliximab and adalimumab concentrations required for mucosal healing in Crohn’s patients were estimated to be in the range 40–80 nM when using a different assay.

All six subjects had high concentrations of active V565 in their faeces after a single oral dose. In all cases the concentrations were in the micromolar range at or around 24 hours after dosing and in one case active drug was detected at the last collection three days after dosing (Fig. 2). The scatter plot shows all measured faecal concentrations between dosing and discharge from the residential unit. The pattern of faecal V565 concentration will be influenced by both gastric release patterns and GI transit times. Post dose faecal samples obtained earlier than the transit time will contain no V565. Likewise, if mini-tablet release from the stomach is synchronous, no V565 would be expected in faecal samples much beyond the transit time. No V565 was detected in the serum (LLOQ = 62.5 ng/ml). In subject 42003 active V565 was detected in the urine collected between 4–8 hours post dose.

### Demonstration of presence and effect of V565 in UC patients after oral dosing

All five patients had biopsies taken before and after dosing for the assessment of both V565 presence in lamina propria and level of phosphorylation. In addition to detection of V565 in post dosing biopsies, further characterisation was carried out to determine co-localisation with both CD3^+^ and CD14^+^ stained cells.

### V565 detection in the colon lamina propria and co-localisation with CD3^+^ and CD14^+^ cells

**Figure 3:**
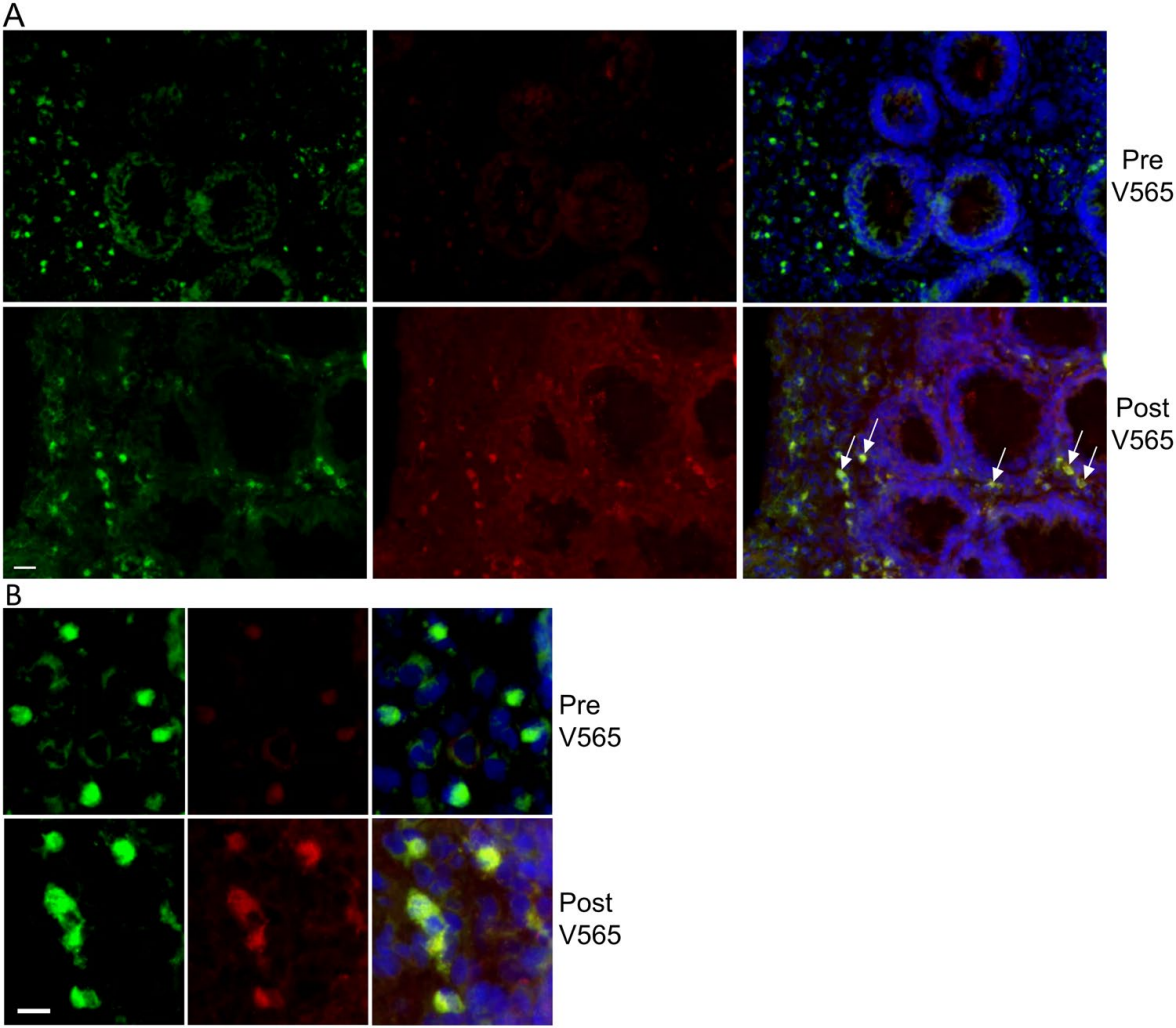
V565 distribution and co-localisation with CD3^+^ cells in colon biopsy sections from ulcerative colitis patients after receiving oral V565 over 7 days. Colon biopsies were taken before and after 7 days of V565 dosing (1665 mg/day) and were embedded in OCT, then snap-frozen. Biopsy sections were cut, fixed and incubated with anti V565 rabbit polyclonal antibody SY7915 and mouse anti-CD3, after which they were washed and incubated with anti rabbit IgG-Alexa 594, anti-mouse-Alexa 488 and Hoechst 33342. V565 associated fluorescence (red) was increased in sections from the post V565 treatment biopsies and some of it co-localised on (green) CD3^+^ cells (arrows and B). Nuclei are blue. Bar = 10 μm and 20 μm in A and B respectively.

**Figure 4:**
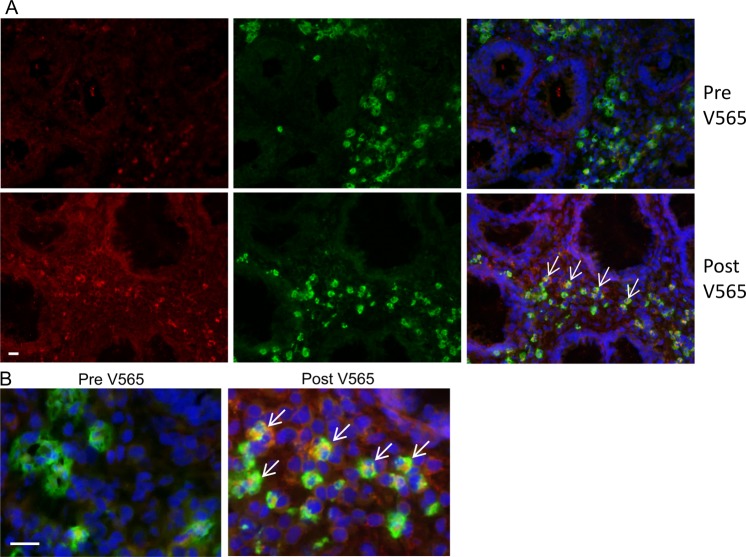
V565 distribution and co-localisation with CD14^+^ cells in colon biopsy sections from ulcerative colitis patients after receiving oral V565 over 7 days. Colon biopsy sections were prepared as in Fig. 3. Sections were incubated with anti-V565 rabbit polyclonal antibody SY7915 and mouse anti-human CD14-FITC. Anti-V565 SY7915 binding was detected with anti rabbit IgG-Alexa 594. Hoechst 33342 was used for nuclear fluorescence, (blue). V565 and CD14 associated fluorescence are red and green, respectively. Bar = 10 μm, Arrows indicate V565 binding on CD14^+^ cells.

Colon biopsy sections were examined for the presence of V565 and also to investigate whether V565 co-localised with CD3^+^ and CD14^+^ cells. Figures 3 and 4 show images from pre- and post-V565 dosing colon biopsy sections in addition to CD3^+^ cells or CD14^+^ cells, respectively. Whilst there was some variation in fluorescence intensities across sections, V565-associated fluorescence was greater in the post-V565 colon biopsy sections, a finding borne out by the fluorescence intensity scores (see below). Moreover, V565 was seen to co-localise with TNFα^+^ CD3^+^ cells in the post-V565 dose biopsy sections. Similarly, V565 was also seen to co-localise with TNFα^+^ CD14 + cells (Fig. 4) in the post V565 biopsy sections.

V565 fluorescence intensity scores were averaged for sections from the pre- and post-V565 dosing biopsies and are shown in Supplementary Fig. S1. All intensity scores were assigned by scientists blinded to the timing of the biopsy in relation to dosing. The mean pre-V565 and post-V565 staining intensity scores were 0.58 ± 0.08 and 1.42 ± 0.18 respectively making the post V565 fluorescence intensity score approximately three-fold higher than the pre-dosing V565 value (p < 0.01, one way ANOVA). The data show that V565 was able to penetrate into the colon tissue of UC patients.

### Measurement of tissue phosphoproteins

**Figure 5:**
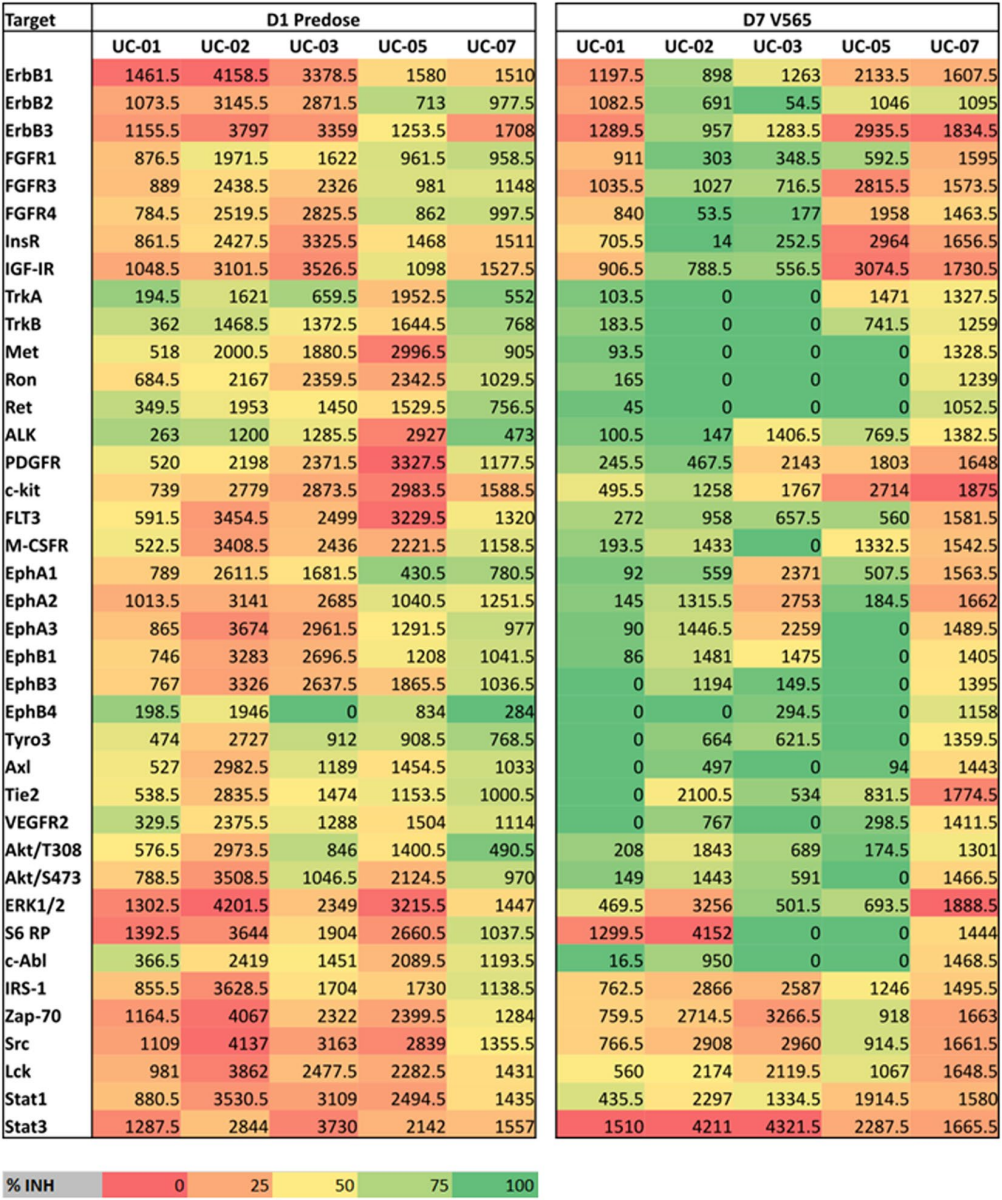
Analysis of tissue phosphoprotein levels in biopsies of ulcerative colitis patients taken before and after 7 days V565 dosing. Relative intensity values of phosphoproteins in the paired pre-dose control and 7d V565 dosed biopsies were compared using conditional formatting in Excel such that the shade of the colour in each cell of the spreadsheet was representative of the phospho-intensity value. The formatted “D1 Predose” and “D7 V565” data for all five UC patients were then grouped. The change from redder to greener shading of the formatted data in the biopsies taken from four patients (UC01, UC02, UC03 and UC05) after dosing reflects inhibition of tissue phosphoprotein levels, while for patient UC07 phosphorylation levels were increased.

**Figure 6:**
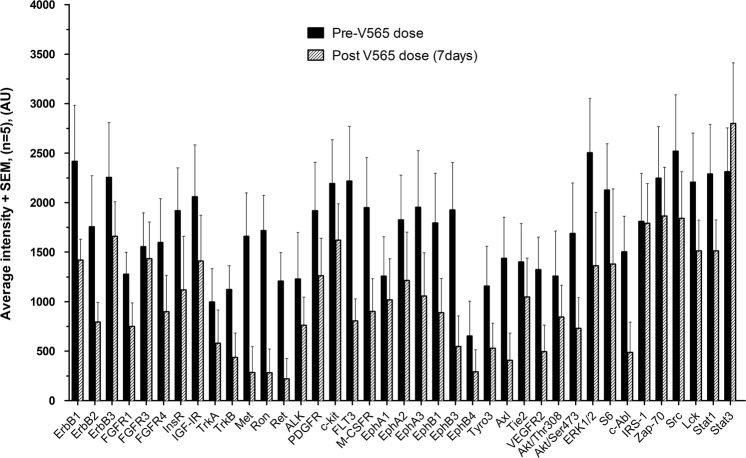
Average intensity levels of individual phosphoproteins in colonic biopsies of pre-dose and after 7d V565 dosing in ulcerative colitis patients. The average phosphoprotein levels (Mean + SEM; n = 5 UC patients) were calculated for all analytes (n = 39) measured in the biopsies taken before (D1 Predose) and after (D7 V565) dosing with V565.

Levels of phosphorylation were analysed in the colon tissue samples taken from each UC patient at baseline and after 7d oral dosing with V565. Analysis of the *ex vivo* cultured pre-dose biopsies taken on Visit 2, showed that incubation with V565 resulted in the inhibition of tissue phosphoprotein levels for each patient by approximately 50% (Supplementary Figs S2 and S3) and confirmed that measurement of this biomarker could be used to provide evidence of TNFα-dependent pharmacological activity in biopsies taken during the *in vivo* dosing phase of the study. For the biopsies taken before and after 7d oral V565, the phosphorylation intensities of individual analytes are presented in Fig. 5. The heatmap (Fig. 5) shows that in four patients (UC01–UC05) the phosphorylation levels of most proteins on the array were decreased (shift from red to green colouration) following dosing with V565, which is consistent with anecdotal reports of improved clinical condition of these subjects. However, for patient UC07 an increased level of phosphorylation (shift to redder colouration) was detected in the biopsy taken after 7d dosing with V565 and this coincided with increased visible ulceration in the second study endoscopy and a reported worsening of clinical symptoms in this individual. The average phosphoprotein levels (Mean + SEM; n = 5 UC patients) calculated for all analytes (n = 39) measured in the biopsies taken before and after V565 dosing are shown in Fig. 6.

**Figure 7:**
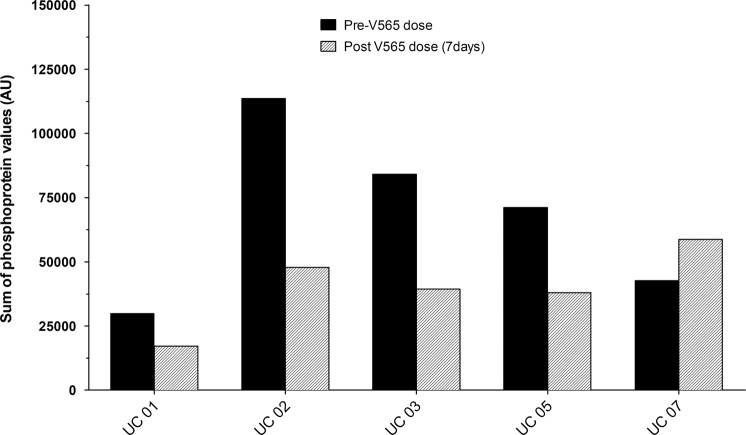
Total phosphorylation levels in biopsies of ulcerative colitis patients taken before andafter 7 days V565 dosing. For each patient, total phosphorylation values for the D1 Predose and D7 V565 biopsies were calculated by summing the signal intensities measured for all 39 of the analytes detected on the phosphoprotein array.

It is not known which, if any of the phosphoproteins detected in this analysis might be more important as a biomarker(s) of disease activity so the effects of dosing on the total protein phosphorylation signal (Σ_n=39_ phosphoproteins) detected for each biopsy was also assessed. As shown in Fig. 7, after dosing with V565 the total phosphorylation levels measured in biopsies from patients UC01-UC05 were inhibited by approximately 50% while the level in patient UC-07 was increased. In this study, the lack of phosphoprotein inhibition observed for one patient following V565 dosing is consistent with the non-response rate of 20–40% that has been reported for anti-TNFα antibodies in the general UC patient population.

### Measurement of serum cytokines

The concentrations of IL-6, TNFα and IL-10 were measured in serum taken before and after dosing using an ultrasensitive immunoassay. Results, both pre- and post-dosing were within or close to the normal range and generally did not show significant changes in either IL-6 or IL-10 (Supplementary Table S2) but a small inhibitory effect of dosing on serum TNFα concentrations was detected for all patients (Supplementary Fig. S4) with mean concentrations in pre-dose and d7 sera of 6.40 ± 1.92 pg/ml and 5.50 ± 2.18 pg/ml, respectively (Mean ± SD; n = 5 patients). However, due to the small sample size and the generally low concentrations measured throughout the study it was not possible to assess the statistical significance of these changes.

### Serum, urine and faecal levels of V565 and ADA titres at day 21

Similar to prior results, all patients provided pre and post dosing serum samples and no V565 was detected in the serum of any of the patient volunteers. V565 was, however, confirmed in the urine of two of the UC patients. In this study the analysis of faecal V565 content was performed on aspirate of luminal contents taken at the time of the post-dosing sigmoidoscopy. Such a sample was obtained from three of the five patients: patients 01, 02 and 07. V565 was detected in the luminal contents of patients 01 and 02, but not 07.

One patient (01) had ADAs at the same titre in the pre-dose and follow up sample. They were not neutralising. No other patient had ADAs in any sample.

### Safety assessment

There were no deaths, serious adverse events, or withdrawals due to adverse events.

All reported adverse events were of mild severity with the exception of one moderate headache in one patient. There were no clinically relevant adverse findings in laboratory test results, vital signs measurements, 12-lead ECG parameters or physical examination results.

## Discussion

Anti-TNFα antibodies are recognised as effective treatments for IBD. The ability to deliver this modality orally to intestinal tract inflammation could be a significant step forward in the treatment available to patients with IBD. The results presented here are therefore encouraging and support the potential utility of V565 in the treatment of IBD patients. In the ileostomy study, the very high V565 concentrations seen in ileostomy bags of all four subjects demonstrate reliable survival in the stomach and small intestine, consistent with the gastro-protective and targeted release characteristics of the mini-tablet formulation. The coating of mini-tablets was designed to provide stability in acid for up to 6 hours, but to allow dissolution and release of active substance over a period of approximately two hours once the pH reached 6.5^19^. The recovery of some partially dissolved mini-tablets in the ileal fluid was therefore to be expected. Mini-tablets were not detected in the stool of patients or volunteers, so the combination of high concentrations of active V565 plus the recovery of mini-tablets in ileal fluid is encouraging as the V565 retained in the mini-tablets at the terminal ileum will be released as the mini-tablets migrate the colon. This, together with the transit of intact V565 released in the upper small intestine, therefore provides a further potential for continued release of V565 through the parts of the intestine most commonly affected by IBD.

V565 has been minimally absorbed following oral administration in all species examined^18^^,^. In patients with Crohn’s disease, where there is increased intestinal permeability^2,3^, it is highly likely that absorption will be increased, and the pharmacokinetic results from the Crohn’s patients provide some valuable insights. Recent studies have shown that systemically administered anti-TNFα agents (infliximab and adalimumab) can pass from the serum into inflamed IBD mucosal tissue^12,13^ and can also pass through the intestinal mucosa into the gut lumen^12,14^. Clearance via this route is consistent with the leakiness of inflamed mucosal epithelium in patients with IBD. We have demonstrated in ileostomy subjects and patients with CD that following a single oral dose of V565, peak concentrations of the antibody were 100–1000 μM in ileal fluids collected between 1–4 h and high concentrations (1–80 μM) were still present in faeces at or around 24 h. These concentrations greatly exceed those reported for the serum levels of adalimumab required for mucosal healing (54–80 nM) and given the evidence for mucosal permeability it is reasonable to argue that redistribution of V565 from the lumen of the intestines into inflamed mucosa could also result in therapeutically effective tissue concentrations of the antibody.

Although no V565 was detected in the serum of any patient, the fact that V565 was detected in the urine of a subject with no features of enterovesical fistula confirms that V565 passed through the lamina propria and into the systemic circulation before being excreted in active form in the urine. This is important in a disease with transmural inflammation. The fact that V565 was only recovered in the urine collection taken between 4 and 8 hours post dose is also potentially important as it reinforces the expectation from preclinical studies that any absorbed V565 will be rapidly excreted. The fact that V565 was recovered from the urine of only one subject most likely reflects differences in inflammation as there was no objective assessment of disease activity prior to enrolment, and all subjects were recorded as having mild disease based on a clinical assessment. The absence of detectable V565 in all serum samples is consistent with the expected minimal absorption and rapid clearance. More detail on absorption and excretion will no doubt accrue from larger studies in populations with more active disease. The confirmation of high faecal V565 concentrations in all subjects at around 24 h after dosing is very encouraging for a suitable dosing regimen for Crohn’s and UC patients.

Finally, the high luminal concentrations of active V565 after oral dosing provides important confirmation that the compound survives well in the intestinal environment, but does not necessarily demonstrate that this confers clinical efficacy. The results of the UC target engagement study are therefore important in demonstrating that V565 enters inflamed tissue and reduces overall phosphorylation of a range of kinases and signalling molecules. V565 co-localisation with CD3^+^ T cells and CD14^+^ macrophages likely reflects binding of V565 to membrane associated TNFα resulting in the inhibition of TNFα signalling. The inhibition of tissue phosphorylation observed in this study therefore provides evidence of pharmacology secondary to the neutralisation of TNFα activity. Given the variability in inflammatory signalling pathways, the important comparison for this outcome is most likely the comparison of overall change within a patient over time. In four of the five patients there was a 50% reduction in overall phosphorylation after 6–7 days oral dosing. This is consistent with an earlier study^18^ where infliximab at a concentration of 10 µg/ml (a serum concentration associated with mucosal healing of IBD^21^) produced a reduction of 50% in the overall phosphorylation in biopsies from four UC patients compared to a control antibody. It is not clear why patient 07 differed in response from the other four patients in this study but there are a number of possible reasons. The coincident increased phosphorylation measured in post-dosing biopsy and worsening of disease in this patient would be consistent with the existence of TNFα-independent inflammation but could also be explained by non-adherence to study medication, given that the *in vitro* incubation with V565 (Supplementary Figs S2 and S3) showed responsiveness to V565, and there was no detectable V565 in the post dosing faecal aspirate.

The absence of detectable V565 in the serum is entirely consistent with the predicted profile of the compound. The recovery from the urine of two subjects with UC is important further confirmation that active V565 passes into and across the lamina propria into the systemic circulation and is excreted into the urine. Quantification of faecal concentrations was not possible in this study as the assay was carried out on luminal aspirates taken after enema at the time of sigmoidoscopy. Two of the five patients had no residual luminal contents to sample. Of the three remaining patients, two revealed detectable faecal V565 while the third (patient 07 – the “non-responder”) did not. Possible reasons for this are discussed above.

There are some limitations of these investigations which deserve comment. The numbers are small and the populations studied were heterogeneous but nevertheless the results on survival within the GI tract and reduction of phosphorylation after oral dosing are encouraging. Dosing periods were short, comprising either single dose for the elucidation of pharmacokinetics or six-seven days for the demonstration of the presence of drug in mucosa and biological effect. These were chosen based on the specific objectives of each study: initial determination of V565 survival in the GI tract in the ileostomy and Crohn’s patients, and demonstration of biological effect in UC patients. The purpose of this latter study was to demonstrate passage of the drug to the distal GI tract, mucosal-absorption and to replicate *in-vitro* data on signalling of inflammatory pathways in the colonic epithelium. As patients were only treated with V565 for a maximum of one week formal clinical scores were not part of the primary or secondary outcomes and were not formally recorded. In addition we deliberately chose patients with only mild, stable disease activity as they were a more reliable group to enrol into a phase 1b trial primarily designed for pharmacodynamics outcomes. There were no adverse or serious adverse events during the study. Anecdotally all but 1 patient described feeling the same or better whilst on the drug. A formal assessment of clinical severity score, endoscopic score and histopathology will be addressed in future assessments of clinical effectiveness. In the UC patients, CRP was measured pre and post dosing but was in the normal range at both time points for all patients. The decision was taken not to collect faecal calprotectin as part of the study because sample handling is critical for reliable results, and this was felt to be logistically difficult and potentially distracting from the primary purpose of the study.

The potential effect of the preparation for sigmoidoscopy on mucosal V565 concentrations was not controlled. The preparation used the usual clinical practice enema at the clinical site and the authors do not expect this would disrupt the colonic epithelium to the extent that it would materially effect mucosal V565 concentrations. Moreover, laboratory tests showed that the contents of the enema used do not interfere with V565 detection by ELISA (data not shown). Finally, the assessment of mucosal presence of V565 was only formally assessed in UC patients, where inflammation is more superficial than in CD. In this context, it is important to note that active V565 has been detected in the urine of patients with both UC and CD after oral dosing. This indicates that V565 will access the transmural inflammation seen in CD. The results of an ongoing large, Phase II study in CD patients will provide more information on this aspect.

## Conclusion

Overall, these studies show that following oral dosing, active V565 reaches IBD disease sites in high concentrations. Furthermore, V565 was shown to bind to its target, TNFα, in UC lesions inducing pharmacological responses. These results are encouraging for the clinical utility of V565 in the treatment of IBD. V565 is currently being studied in a large, placebo controlled study in Crohn’s disease which should further elucidate its clinical profile (NCT02976129).

## Supplementary information

Supplementary Information

## Data Availability

All data generated or analysed during this study are included in this published article (and its Supplementary Information Files).
