# Supplementary material for: Oral Anti-Tumour Necrosis Factor Domain Antibody V565 Provides High Intestinal Concentrations, and Reduces Markers of Inflammation in Ulcerative Colitis Patients
Source: Sci Rep. 2019 Oct 1;9:14042. doi: 10.1038/s41598-019-50545-x (PMC6773840; doi:10.1038/s41598-019-50545-x)
Supplement: Supplementary file 1 — Supplementary Information [file 41598_2019_50545_MOESM1_ESM.pdf]

SUPPLEMENTARY INFORMATION

Oral Anti-Tumour Necrosis Factor Domain Antibody V565 Provides High Intestinal Concentrations, and Reduces Markers of Inflammation in Ulcerative Colitis Patients

Authors: Suhail Nurbhai*,[1] Kevin J. Roberts,[2] Timothy M. Carlton,[2] Luana Maggiore,[2] Marion F. Cubitt,[2] Keith P. Ray,[1] Jill Reckless,[3] Hafeez Mohammed,[3] Peter Irving,[4] Thomas T. MacDonald,[5] Anna Vossenkämper,[5] Michael R. West,[1] Gareth C. Parkes[6] and J. Scott Crowe[1,2]

1. VHsquared Ltd., 1 Lower Court, Copley Hill, Cambridge Road, Babraham, Cambridge, CB22 3GN, UK
2. VHsquared Ltd., Wellcome Sanger Institute, Wellcome Genome Campus, Hinxton, CB10 1SA, UK
3. RxCelerate Ltd. Babraham Research Campus, Cambridge CB22 3AT, UK
4. Guy's and St Thomas' Hospital, London, UK
5. Blizard Institute, Barts and the London School of Medicine and Dentistry, Queen Mary University of London, London, UK
6. Royal London Hospital, London, UK

Supplementary Table S1: Characteristics of UC patients enrolled in this study.

Patient	Disease Duration	Baseline Mayo	UC medication
01	1.6y	3	Pentasa 2g/d since Dec 2016
02	10.2y	7	Mezavant 4.8g/d since May 2007<br>Mercaptopurine 150mg/d since Nov 2010
03	10.1y	4	Mesalazine 1.2g/d since Jul 2007<br>Azathioprine 150mg/d since Jun 2008
05	1.4y	6	None
07	23.3y	6	Octasa 2.4g/d since Sep 2017 (1.6g/d from 1994 – Sep 2017)

Supplementary Table S2: Serum cytokine concentrations in samples taken from ulcerative colitis patients before and after 7 days V565 dosing.

		Cytokine Concentration (pg/ml)	
**Subject**	Analyte	Visit 2	Visit 3
**01_01**	IL-6	1.22	0.80
**01_02**	IL-6	11.69	6.31
**01_03**	IL-6	4.31	4.81
**01_05**	IL-6	2.74	0.81
**01_07**	IL-6	2.22	1.67
**01_01**	TNFα	5.94	4.42
**01_02**	TNFα	7.08	6.24
**01_03**	TNFα	9.27	8.89
**01_05**	TNFα	5.62	4.73
**01_07**	TNFα	4.11	3.23
**01_01**	IL-10	0.86	0.87
**01_02**	IL-10	2.21	2.06
**01_03**	IL-10	11.34	10.57
**01_05**	IL-10	3.17	2.75
**01_07**	IL-10	0.36	0.60

The concentrations of IL-6, TNFα and IL-10 in serum samples taken before (Visit 2) and after (Visit 3) oral V565 dosing were determined at the Immunoassay Biomarker Core Laboratory, University of Dundee, on a Simoa HD-1 analyser using the Quanterix™ Simoa Human Cytokine 3-plex A according to the manufacturer's instructions. The results obtained for all five UC patients are shown.

Supplementary Figure S1: V565 mean fluorescence intensity scores on sections of ulcerative colitis patients' colon biopsies taken before and after 7 days V565 dosing.

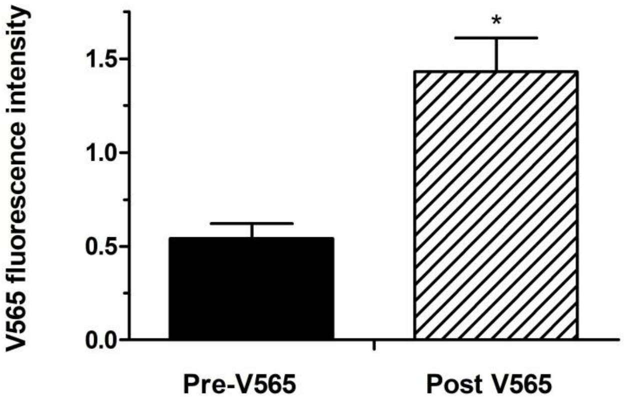

Colon biopsy section images (taken under identical conditions) were scored blindly for V565 fluorescence intensity. Data are V565 fluorescence intensity means and standard errors for patients' colon biopsy sections pre- and post V565 dose. *p< 0.01 (one way ANOVA).

Supplementary Figure S2: Analysis of tissue phosphoprotein levels in pre-dose biopsies of ulcerative colitis patients following 24h organ culture with V565 or a matched ID2A control antibody.

A)

Target	Organ Culture - Control Treated					Organ Culture - V565 Treated				
	UC-01	UC-02	UC-03	UC-05	UC-07	UC-01	UC-02	UC-03	UC-05	UC-07
ErbB1	1627	4165.5	3403.5	1393.5	791.5	2039.5	48.5	13	876.5	767
ErbB2	1273	2771	2506	649	442.5	1704	90.5	96	0	122
ErbB3	1110.5	2369.5	2436.5	1683.5	1301.5	1847	546	1099	2260	895
FGFR1	926	4437.5	2820	1260.5	584.5	780.5	41.5	1362	0	338.5
FGFR3	1000	3734	2966	921	889	1098.5	602	1093	328.5	507
FGFR4	758	3083	2684.5	1531.5	714	932.5	94.5	294.5	577.5	421.5
InsR	633.5	2293.5	2365.5	1704.5	1084	719	288.5	0	1963.5	659.5
IGF-IR	280	1910.5	2068.5	1938	1239	727.5	887.5	93	1845.5	797
TrkA	144	2580	1260	563	383.5	172	245.5	0	52.5	119.5
TrkB	205	2329	1428	624.5	181.5	216.5	178	0	0	0
Met	194	3316.5	1562.5	2035.5	113	70	100	0	0	0
Ron	222.5	2847	1259.5	2841	199.5	126.5	288.5	0	0	30
Ret	3	1918.5	96	1786.5	0	44	152.5	0	0	0
ALK	224	2814.5	656	1966.5	292.5	0	89.5	907	0	0
PDGFR	215.5	3588	1496	2181	524	0	67.5	1387.5	0	248.5
c-kit	212.5	3793.5	1453	2618.5	1026	0	542.5	1590	717.5	730.5
FLT3	0	3801	1026.5	2665	454	0	14.5	1106	0	219
M-CSFR	10.5	3359	694.5	2109.5	343.5	0	689	769	677	458.5
EphA1	474.5	2889.5	1679	1615.5	289	18	0	898.5	26.5	19.5
EphA2	360.5	4104.5	2122	2048	462.5	0	0	1443.5	0	84
EphA3	525.5	4034	1859.5	1849.5	334.5	0	122.5	1469	0	76.5
EphB1	168	3662.5	1332	943	399	0	0	1332.5	0	145.5
EphB3	110	3215	847	970	153	0	0	1009	0	125.5
EphB4	0	617	5.5	1069	22	0	0	0	0	0
Tyro3	141	2664	952	1631.5	373	0	157.5	0	46	37
Axl	184.5	2700	1026.5	1609	534	0	0	0	140.5	209.5
Tie2	217.5	2680.5	1428.5	1842	883.5	101	1956	883	388	610.5
VEGFR2	0.5	1749.5	395.5	819.5	567.5	0	520	0	0	448
AktT-308	437.5	1823.5	763.5	2918.5	116.5	144.5	962.5	0	355.5	0
Akt S-473	287	2248	1229.5	2765.5	290	84.5	593	0	4.5	105
ERK1/2	578	3057	2262.5	3159.5	723.5	751.5	2757	0	1296.5	528.5
S6 RP	1460	2460	1880.5	2409	678.5	1458.5	3900.5	0	191	521.5
c-Abl	452.5	2223.5	535.5	1485	654.5	207.5	777.5	0	788	388
IRS-1	663	2098	1710.5	2913.5	473.5	643.5	885	878	2366.5	391
Zap-70	1064	2694	2274	3048	334.5	385	803	1381.5	1735.5	285.5
Src	1122	3351.5	2295	3202.5	530.5	567.5	816	1313.5	1697.5	426
Lck	1013	3225	2103.5	2743	845	721.5	725.5	1358.5	2226.5	434
Stat1	1015	3550.5	1699.5	2504	989.5	746.5	1521	1489.5	1807	332.5
Stat3	1375.5	2098	3023.5	2469.5	983.5	1354.5	781.5	3802	3141	817

% INH	0	25	50	75	100

B)

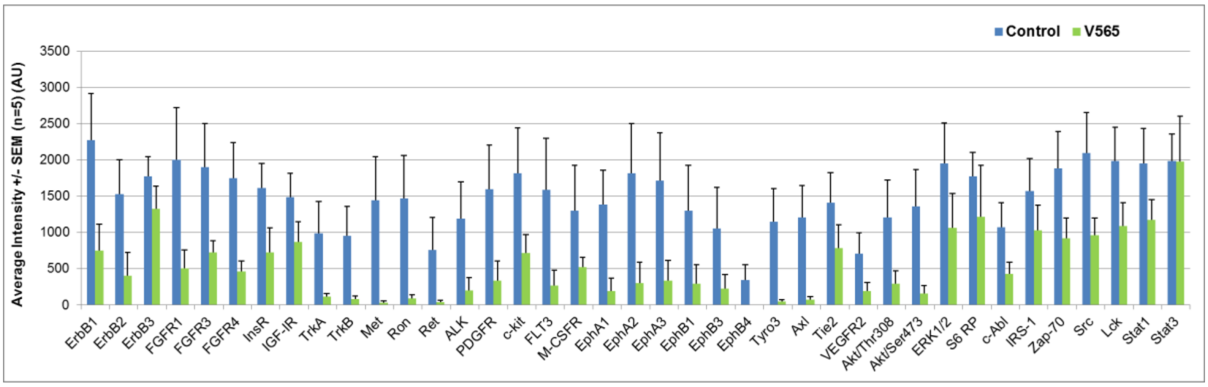

Pre-dose biopsies taken from each ulcerative colitis patient on Visit 2 were maintained in organ culture for 24h with the addition of ID2A (matched control VHH) or V565 each at 150 nM. The tissue collected from each well at the end of the incubation period was snap-frozen and the phosphorylation status of 39 receptor tyrosine kinases (RTK) and signalling molecules analysed on PathScan RTK signalling arrays.

Figure S2A: Intensity values of the phosphoproteins measured in the paired ID2A control and V565 treated biopsies were compared using conditional formatting in Excel such that the shade of the colour in each cell of the spreadsheet was representative of the phospho-intensity value. Formatted control and V565 data for all five UC patients were then grouped according to treatment. The change from redder to greener shading of the formatted data in the biopsies cultured with V565 reflects inhibition of tissue phosphoprotein levels. Figure S2B: Average phosphoprotein levels (Mean + SEM; n=5 UC patients) were calculated for all analytes (n=39) measured in the biopsies following 24h incubation with either the control antibody (blue) or V565 (green).

Supplementary Figure S3: Total phosphorylation levels in pre-dose biopsies of ulcerative colitis patients following organ culture with V565 or a matched control antibody.

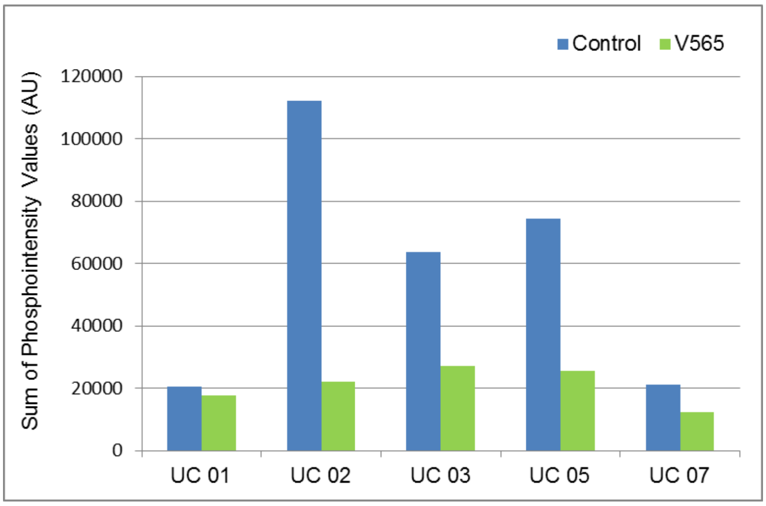

Pre-dose biopsies taken from each ulcerative colitis patient on Visit 2 were maintained in organ culture for 24h with the addition of ID2A (matched control VHH) or V565 each at 150 nM. The tissue collected from each well at the end of the incubation period was snap-frozen and the phosphorylation status of 39 receptor tyrosine kinases (RTK) and signalling molecules analysed on PathScan RTK signalling arrays. Total phosphorylation values for the biopsies cultured with the control antibody (blue) or V565 (green) were calculated by summing the signal intensities measured for all of the analytes. Values are shown for all five UC patients.

Supplementary Figure S4: Serum concentrations of TNFα in samples taken from ulcerative colitis patients before and after 7 days V565 dosing.

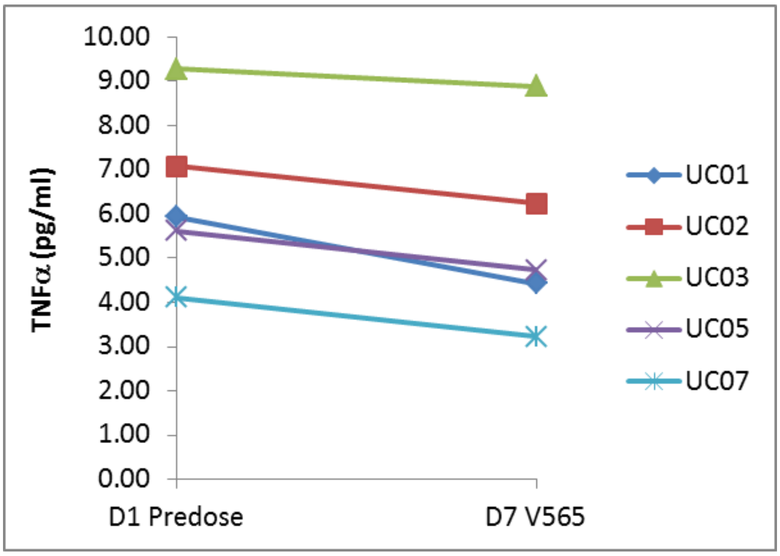

Serum samples for the measurement of TNFα were collected prior to dosing at Visit 2, and after the final dose at Visit 3. The concentrations TNFα were determined using high sensitivity QuanterixTM Simoa assay technology according to the manufacturer’s instructions.
